# Prevalence and Clinical Characteristics of Hearing Loss Caused by *MYH14* Variants

**DOI:** 10.3390/genes12101623

**Published:** 2021-10-15

**Authors:** Ken Hiramatsu, Shin-ya Nishio, Shin-ichiro Kitajiri, Tomohiro Kitano, Hideaki Moteki, Shin-ichi Usami, on behalf of the Deafness Gene Study Consortium

**Affiliations:** 1 Department of Otorhinolaryngology, Shinshu University School of Medicine, 3-1-1 Asahi, Matsumoto, Nagano 390-8621, Japan; matsuken@shinshu-u.ac.jp (K.H.); nishio@shinshu-u.ac.jp (S.-y.N.); kitajiri@shinshu-u.ac.jp (S.-i.K.); tomokitano@shinshu-u.ac.jp (T.K.); moteki@shinshu-u.ac.jp (H.M.); 2 Department of Hearing Implant Sciences, Shinshu University School of Medicine, 3-1-1 Asahi, Matsumoto, Nagano 390-8621, Japan

**Keywords:** *MYH14*, nonsyndromic hearing loss, DFNA4, postlingual onset hearing loss, late-onset hearing loss, progressive hearing loss, massively parallel DNA sequencing

## Abstract

Variants in *MYH14* are reported to cause autosomal dominant nonsyndromic hereditary hearing loss (ADNSHL), with 34 variants reported to cause hearing loss in various ethnic groups. However, the available information on prevalence, as well as with regard to clinical features, remains fragmentary. In this study, genetic screening for *MYH14* variants was carried out using a large series of Japanese hearing-loss patients to reveal more detailed information. Massively parallel DNA sequencing of 68 target candidate genes was applied in 8074 unrelated Japanese hearing-loss patients (including 1336 with ADNSHL) to identify genomic variations responsible for hearing loss. We identified 11 families with 10 variants. The prevalence was found to be 0.14% (11/8074) among all hearing-loss patients and 0.82% (11/1336) among ADNSHL patients. Nine of the eleven variants identified were novel. The patients typically showed late-onset hearing loss arising later than 20 years of age (64.3%, 9/14) along with progressive (92.3%, 12/13), moderate (62.5%, 10/16), and flat-type hearing loss (68.8%, 11/16). We also confirmed progressive hearing loss in serial audiograms. The clinical information revealed by the present study will contribute to further diagnosis and management of *MYH14*-associated hearing loss.

## 1. Introduction

Hearing loss (HL) is the most common sensory impairment. A wide variety of genes and variants have been reported as causative for HL, and more than 120 genes have been reported to cause nonsyndromic HL [1]. The clinical features of HL, including age at onset, progression, severity, audiometric configuration, and effectiveness of interventions, differ among the various genes and variants [2]. Toward the more appropriate treatment of each HL patient, the identification of the causative variants by genetic testing and clarification of the clinical characteristics caused by each deafness gene variant are vital.

The inheritance patterns of hereditary HL include autosomal recessive, autosomal dominant, X-linked, and mitochondrial patterns. A majority of autosomal dominant nonsyndromic hereditary hearing loss (ADNSHL) is postlingual onset, progressive HL, which accounts for approximately 20% of nonsyndromic hereditary HL patients [2]. To date, 46 causative genes for ADNSHL have been identified [1].

*MYH14* encodes nonmuscle myosin II C (NMIIC), a member of the myosin superfamily, and is a causative gene for ADNSHL (DFNA 4) [3]. It is widely expressed in the inner ear, including the organ of Corti. There have been reports of 34 *MYH14* variants causing ADNSHL [4,5,6,7,8,9,10,11,12,13,14,15,16,17,18,19,20,21,22,23,24].

With regard to prevalence, four studies have investigated the prevalence of *MYH14* variants in deafness cohorts from Europe, the USA, and China [4,8,9,10], but the information regarding clinical characteristics, including age at onset, progression, and audiometric configurations, remains fragmentary. In this study, we performed screening for *MYH14* variants for a large number of Japanese HL patients (*n* = 8074, including 1336 with ADNSNL), and examined the clinical features in detail.

## 2. Materials and Methods

### 2.1. Subjects

A total of 8074 Japanese HL patients were enrolled nationwide, as previously reported [25]. All patients showed sensorineural HL, with 1336 of them showing autosomal dominant (AD) inheritance patterns. This study was approved by the Shinshu University Ethical Committee, as well as the respective ethical committees of the other participating institutions, and was conducted in accordance with the Declaration of Helsinki. Informed consent was obtained from all patients (or from their next of kin, caretaker, or legal guardian in the cases of minors or children). Clinical information and peripheral blood samples were obtained from patients and all relatives from whom written informed consent was obtained.

### 2.2. Variant Analysis

Massively parallel DNA sequencing (MPS) analysis for 68 target deafness genes (Table S1) was performed for all patients. The detailed protocol was described elsewhere [25]. An Ion AmpliSeq Custom Panel (ThermoFisher Scientific, Waltham, MA, USA) was designed using an Ion AmpliSeq Designer, and the amplicon libraries were prepared using an Ion AmpliSeq library kit version 2.0 (ThermoFisher Scientific, Waltham, MA, USA). The emulsion PCR and MPS were performed using an Ion PGM, Ion Proton or IonS5 sequencer (ThermoFisher Scientific, Waltham, MA, USA), and the sequence data were mapped against the human genome sequence (build GRCh37/hg19).

The protein-affecting variants (including the missense, nonsense, insertion/deletion, and splicing variants) with an allele frequency of less than 1% of the ExAC03 [26], ToMMo 3.5KJPN [27], and the 333 in-house Japanese normal hearing controls were selected. The annotation for each variant was analyzed by ANNOVAR software ver. 20191024 [28]. Functional in silico predictions were performed for missense variants by SIFT [29], PolyPhen2 [30], Mutation Taster [31], Mutation Assessor [32], FATHMM [33], and Combined Annotation Dependent Depletion (CADD) [34] software programs including in dbNSFP ver.3.5. The remaining *MYH14* variants were confirmed by direct sequencing. Segregation analysis for family members was also performed by direct sequencing. The pathogenicity of the identified variants was evaluated using the American College of Medical Genetics (ACMG) standards and guidelines [35].

The variants classified as “Likely Pathogenic” or “Pathogenic” were considered to be causative variants. In addition, variants classified as being of “Uncertain significance” were also considered to be pathogenic if all three of the following conditions were satisfied: (1) no other candidate variants were identified in the other 67 genes; (2) the allele frequency was under 0.0001 in the control populations in ExAC03, gnomAD, ToMMo 3.5KJPN, and in-house controls; and (3) the CADD score was 20 or more.

### 2.3. Clinical Evaluation

Clinical information, including: (1) onset age; (2) progression of HL; (3) pedigree; (4) episodes or symptoms of vertigo; and (5) intervention for HL, was collected from a review of medical charts. Evaluation of HL was performed by pure-tone audiometry on patients aged 4 years or older, and the auditory steady-state response (ASSR) or play audiometry was performed for those who could not be evaluated by pure-tone audiometry. The pure-tone average (PTA) was calculated from the audiometric thresholds at four frequencies (500, 1000, 2000, and 4000 Hz). The severity of HL was classified into 4 categories: mild (PTA 20–40 dB), moderate (41–70 dB), severe (71–90 dB), and profound (>91 dB) [36]. The audiometric configurations were categorized into Flat, Low-frequency ascending, Mid-frequency U-shaped, High-frequency gently sloping, and High-frequency steeply sloping, as reported previously [36]. Caloric testing was performed for one of the two patients who complained of vestibular symptoms.

## 3. Results

**Figure 1:**
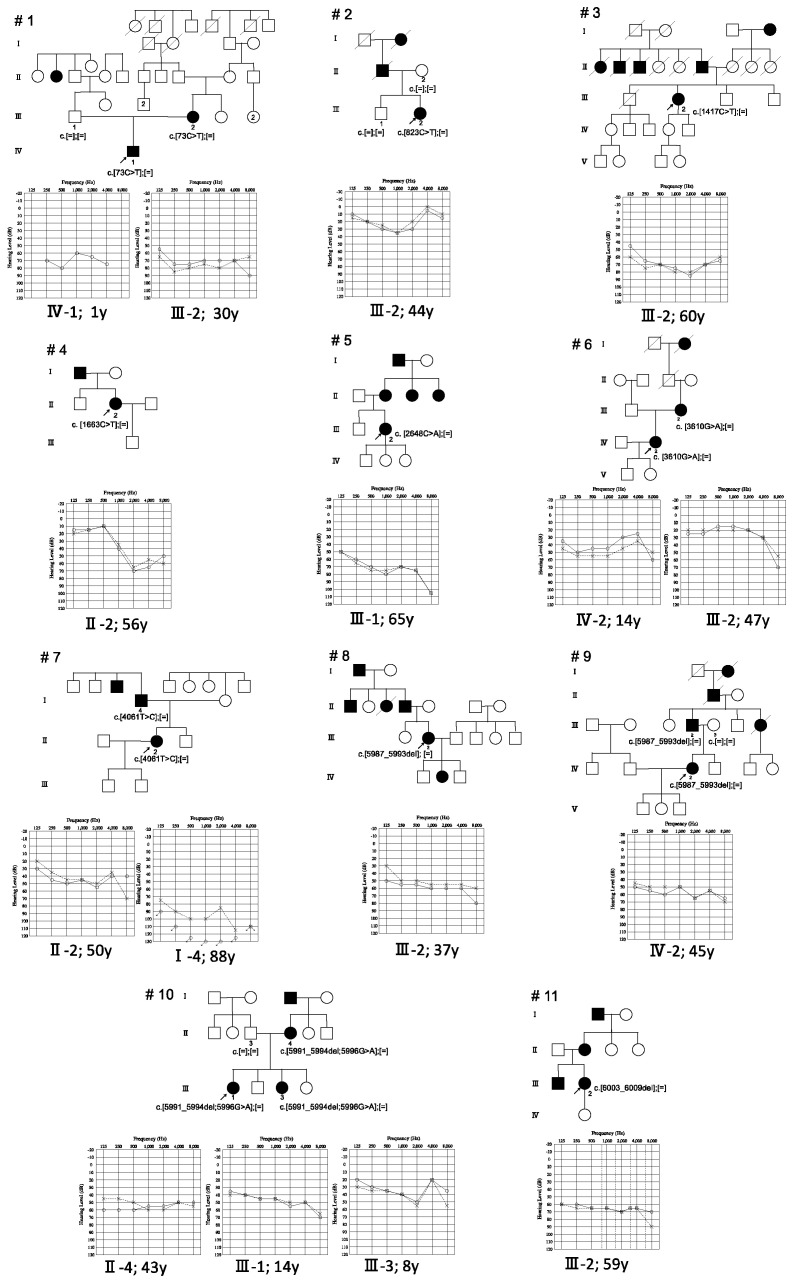
Pedigree, identified variants, and audiograms for the *MYH14*-associated hearing-loss patients identified in this study. Arrowheads indicate the proband for each family. Identified variants are indicated on the pedigree. Audiograms for the proband and other affected family members are shown with the age at hearing testing.

Among the 1336 unrelated ADNSHL probands, we identified 11 probands (Table 1, Figure 1) carrying 10 possibly disease-causing *MYH14* variants (Table 2). Among the 10 variants, 8 were novel. Therefore, the frequency of *MYH14*-related HL patients among Japanese ADNSHL patients was 0.82% (11/1336). Most of the *MYH14* variants in previous reports were missense variants, with only two being nonsense variants (Table 3).

**Figure 2:**
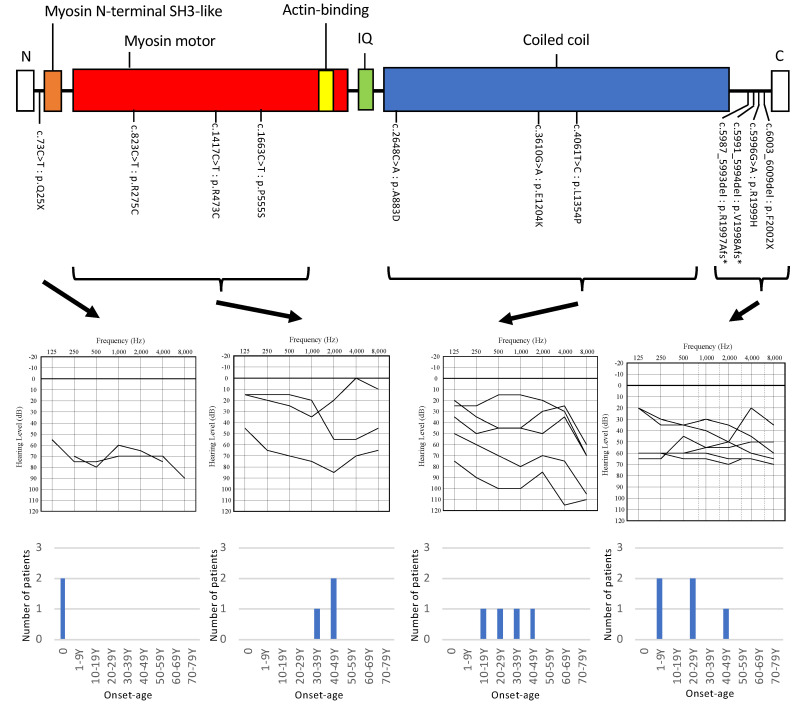
Domain structure of MYH14, identified variants, the overlapping audiograms from the better-hearing ear, and onset-age distribution for the patients with each domain variant. Domain structures are indicated based on UniProtKB (Q7Z406). Orange: Myosin N-terminal SH3 like domain, Red: Myosin motor domain, Yellow: Actin binding domain, Green: IQ domain, Blue: Coiled coil domain.

In this study, one nonsense variant, one frameshift variant, one non-frameshift variant causing a nonsense codon, six missense variants, and one variant with frameshift and missense variants in the *cis* allele were identified. One nonsense variant was located near the N-terminal region of MYH14, three missense variants were located in the Myosin head domain, three missense variants were located in the coiled-coil domain, and the other three variants were located in the C-terminal region (Table 2, Figure 2).

We included the 11 probands and six affected family members for clinical characteristics analysis. Regarding the onset of HL among the 11 probands and six family members identified, most of the patients showed late-onset hearing loss, and only two patients showed congenital onset (Figure 2). Both congenital HL patients had *MYH14* variants located near the N-terminal region (Figure 2). The onset ages for the late-onset HL cases ranged from 3 to 44 (Table 1, Figure 2).

The audiometric configurations of the 11 probands and their affected family members were categorized into Flat (*n* = 11), Mid-frequency U-shaped (*n* = 2), High-frequency gently sloping (*n* = 1), High-frequency steeply sloping (*n* = 1), and Low-frequency ascending (*n* = 1) (Figure 1, Table 1). We could not identify any genotype–phenotype correlation among the domains (Figure 1 and Figure 2). The severity of deafness also varied among cases. In the PTA calculated from the audiometric thresholds at four frequencies (500, 1000, 2000, and 4000 Hz), 3 patients showed mild HL, 10 moderate HL, 2 severe HL, and 1 profound HL (Table 1). However, the one profound hearing loss case in Family No. 7 (Case I-4) had a history of repeated bilateral otitis media since childhood, and he had undergone bilateral middle ear surgery. After this middle ear surgery, he suffered bilateral deterioration in hearing. Thus, it is unclear whether the profound HL observed for this case was purely due to genetic causes. Among the 17 patients, at least 8 patients used hearing aids. Two of the eleven probands complained of dizziness, and one (Family No. 5) underwent an examination for nystagmus and caloric testing, but no obvious abnormal findings were observed.

**Figure 3:**
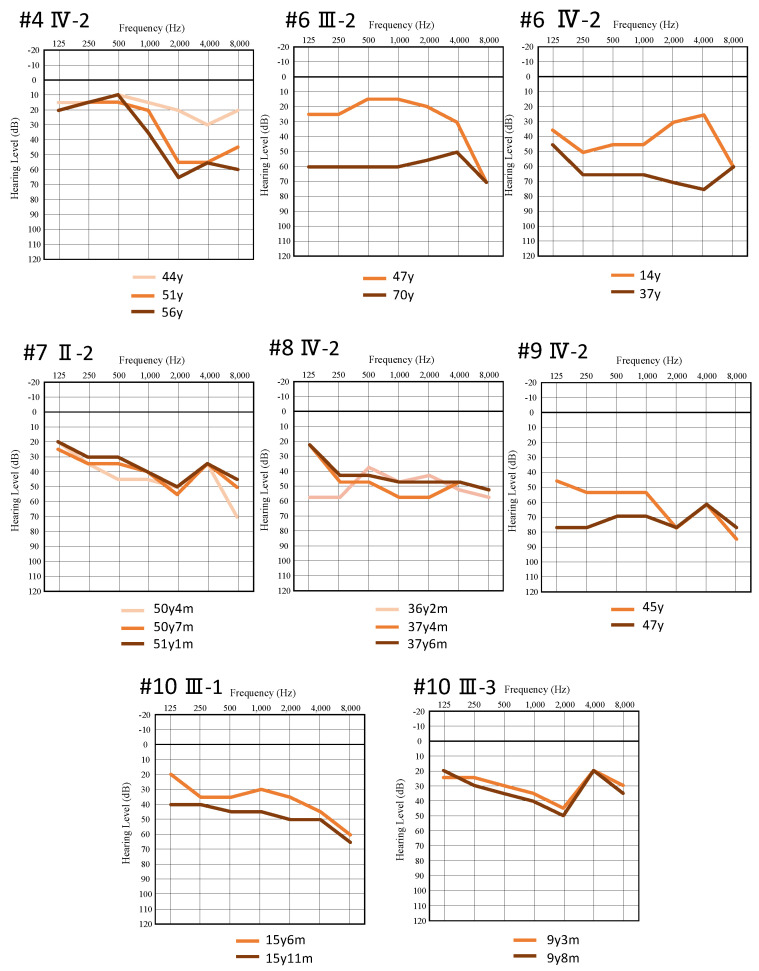
Serial audiograms of eight individuals from six families with *MYH14* variants.

Anamnestic evaluation of the 11 probands and 6 of their family members was also performed, and 12 were conscious of the progression of deafness (Table 1). Figure 3 shows serial audiograms for eight patients from six families. The audiograms for the better-hearing ear were used to evaluate progression. The patients who were observed for more than 10 years showed clear progression (Family No. 4 IV-2, Family No. 6 III-2, and Family No. 6 IV-2). Family No.9 IV-2 also showed hearing progression over two years. The observation periods for Family No. 7, No. 8, and No. 10 were too short to allow evaluation of progression (5 months to 16 months).

## 4. Discussion

As *MYH14*-associated HL is rare, the currently available information regarding the variant spectrum and clinical characteristics is limited. In this study, using a cohort of 8074 HL patients, we identified nine novel variants, and were able to summarize the variant spectrum. This is the largest cohort studied for *MYH14*-associated hearing loss to date. In addition, we were able to clarify the prevalence of *MYH14* gene variants in patients; that is, 0.14% (11/8074) among HL patients and 0.82% among ADNSHL patients (11/1336). To date, there have been four reports of the variant prevalence of *MYH14*-associated HL. Shearer et al. [7] reported that the frequency of *MYH14*-associated HL was 3.0% among all HL patients in the USA (3/100 probands). Sloan-Heggen et al. [8] reported that 5 of 1119 HL patients (0.45%) carried *MYH14* candidate pathogenic variants, and these variants accounted for 3.5% of ADNSHL patients (5/141). Chen et al. [9] reported the prevalence of *MYH14*-associated HL in Chinese to be 2.59% (3/116). It is difficult to compare prevalence, as it depends on study subjects and pathogenicity classification methods. In this study, as stated in Section 2.2, more definitive criteria for pathogenicity were applied.

Various genes have been reported to be causative in ADNSHL families [6,8]; however, at present, there is no particular major responsible deafness gene for ADNSHL. Our screening using the same cohort clarified the frequencies for the other causative genes in ADNSHL patients; *KCNQ4*: 6.6% [37], *POU4F3*: 4% [38], *TECTA*: 2.9% [39], *WFS1*: 2.5% [40], *MYO6*: 2.4% [41], *ACTG1*: 1.1% [42], and *EYA4*: 0.9% [43]. Although *MYH14*-associated HL is rare, the present results indicated that this gene should be included in HL screening, especially that for ADNSHL.

Among the 11 probands, two cases (Family No. 8 and 9) carried the same variant. These two cases were in unrelated families. As the mechanism of these commonly observed variants could have occurred by founder mutation or in a mutational hotspot, haplotype analysis could afford a method of clarifying their genesis. However, we obtained only the proband sample for Family No. 8, and could not perform haplotype analysis.

With regard to onset age, a majority of patients were shown to be have experienced adult onset occurring later than 20 years of age (9/14, 64.3%). The onset age had not been reported previously, except for two pedigrees with congenital or prelingual onset (Table 3). In this study, we clearly determined that late onset was one of the characteristic clinical features of *MYH14*-associated HL. It was noteworthy, however, that there were four patients with an onset age under 10 years old. Among them, HL in one patient (Family No. 1, III-2) was found through the newborn hearing screening program. It is interesting to note that this case carried the same nonsense variant (c.73C > T p.Q25X) as that previously reported in a congenital HL case^4^. This variant was located near the N-terminal region. As all other variants located in the other domains identified in this study did not cause congenital HL, truncation within this domain may cause congenital HL through haploinsufficiency.

Information regarding the severities and audiometric configurations of *MYH14*-associated HL in previous reports was also fragmentary (Table 3). The present study showed that a majority of patients exhibited moderate (62.5%, 10/16) and Flat-type HL (68.8%, 11/16) (Figure 1, Table 1).

In terms of the progression of *MYH14*-associated HL, 92.3% of patients (12/13) had noticed the progression of their HL (Table 1). In this study, as shown in Figure 3, at least five patients showed progression based on serial audiometric evaluations. In particular, three patients (Family No. 4 IV-2, Family No. 6 III-2, and Family No. 6 IV-2) underwent serial audiometric evaluations over more than 10 years and showed obvious progression of HL. The present data strongly supported the notion that the progression of HL, which has been previously reported [3,4], is one of the characteristic features of *MYH14*-associated HL.

With regards to intervention, 8 of the 16 patients used hearing aids (HAs) (Table 1), indicating that hearing aids should be recommended as hearing devices. In this study, no patient received cochlear implantation, suggesting that the HL was within the hearing range for which HAs are indicated in most cases. Liu et al. [15] reported a case in which cochlear implantation was performed for a patient with a *MYH14* variant. However, it should be noted that this case also had a *MYO15A* compound heterozygous variant. Therefore, it is unclear whether the *MYH14* variant itself causes profound hearing loss for which cochlear implantation is indicated.

We could not identify any genotype–phenotype correlations among the domains. In addition, we could not identify any genotype–phenotype correlations through combination of the clinical information obtained in our study and that of previously reported cases.

## 5. Conclusions

The present study revealed an updated variant spectrum and the clinical characteristics of *MYH14*-associated HL, including onset age, severity and progression of hearing loss, audiometric configuration, and recommended intervention. The information provided in this paper will play a crucial role in managing patients in the future.

## Figures and Tables

**Table 1:** Variants and clinical information for *MYH14*-associated HL patients and affected family members identified in this study (all *MYH14* variants are indicated in NM_001145809.2).

Nucleotide Change	Amino Acid Change	Gender	Onset	Progression of HL	Age	Audiometric Configuration	PTA (Better-Hearing Ear, dB)	Severity	Vestibular Symptoms	Intervention	Newborn Hearing Screening	Family No.	Patient No.
c.73C > T	p.Q25X	F	0 s	No	30 Y	Flat	71.25	Severe	No	HA	refer	1	Ⅲ-2
c.73C > T	p.Q25X	M	0	n/a	1 Y	Flat	70	Moderate	n/a	n/a	n/a	1	Ⅳ-1
c.823C > T	p.R275C	F	41 Y	Yes	44 Y	U-shaped	20	Mild	No	n/a	n/a	2	Ⅲ-2
c.1417C > T	p.R473C	F	35 Y	Yes	60 Y	Flat	75	Severe	n/a	n/a	n/a	3	Ⅲ-2
c.1663C > T	p.P555S	F	44 Y	Yes	56 Y	Steeply sloping	41.25	Moderate	No	HA	n/a	4	Ⅱ-3
c.2648C > A	p.A883D	F	44 Y	n/a	65 Y	Gently sloping	73.75	Moderate	Yes	n/a	n/a	5	Ⅲ-1
c.3610G > A	p.E1204K	F	12 Y	Yes	14 Y	Low-frequency ascending	47.5	Moderate	No	HA	n/a	6	III-1
c.3610G > A	p.E1204K	F	n/a	Yes	47 Y	Flat	20	Mild	No	n/a	n/a	6	Ⅱ-3
c.4061T > C	p.L1354P	M	20 s	Yes	83 Y	Flat	100	Profound	n/a	n/a	n/a	7	I-4
c.4061T > C	p.L1354P	F	30 s	Yes	50 Y	Flat	43.75	Moderate	No	HA	n/a	7	II-2
c.5987_5993del	p.R1997Afs *	F	20 Y	Yes	37 Y	Flat	53.75	Moderate	Yes	HA	n/a	8	Ⅲ-2
c.5987_5993del	p.R1997Afs *	F	20 s	Yes	45 Y	Flat	55	Moderate	No	HA	n/a	9	Ⅳ-2
c.5987_5993del	p.R1997Afs *	M	n/a	n/a	n/a	n/a	n/a	n/a	n/a	n/a	n/a	9	Ⅲ-2
c.[5991_5994del; 5996G > A]	p.[V1998Afs *; R1999H]	F	n/a	n/a	43 Y	Flat	55	Moderate	n/a	n/a	n/a	10	Ⅱ-4
c.[5991_5994del; 5996G > A]	p.[V1998Afs *; R1999H]	M	6 Y	Yes	14 Y	Flat	47.5	Moderate	No	HA	pass	10	Ⅲ-1
c.[5991_5994del; 5996G > A]	p.[V1998Afs *; R1999H]	F	3 Y	Yes	8 Y	U-shaped	29	Mild	No	HA	pass	10	Ⅲ-3
c.6003_6009del	p.F2002X	F	41 Y	Yes	59 Y	Flat	66.25	Moderate	No	n/a	n/a	11	Ⅲ-2

* indicate the stop codon as in standrd HGVS nomenclature. n/a, not available; HA, hearing aids; CI, cochlear implant. Family No. and Patient No. indicate the patient number in Figure 1.

**Table 2:** Identified *MYH14* variants and in silico predication score (all *MYH14* variants are indicated in NM_001145809.2).

Nucleotide Change	Exon	Amino Acid Change	Domain	SIFT	PP2	Mut Taster	Mut Assessor	Revel	CADD Phred	Allele Frequency in In-house Controls	MAF in ExAC03	MAF in ToMMo (4.7kJPN)	ACMG Criteria	Reference
c.73C > T	2	p.Q25X	Myosin, N-terminal			A			36	0	0	0	Likely Pathogenic	Donaudy et al., 2004 [3]
c.823C > T	8	p.R275C	Myosin head, motor domain	D	D	D	D	0.648	34	0	0.000008252	0	Uncertain Significance	Iwasa et al., 2016 [6]
c.1417C > T	13	p.R473C	Myosin head, motor domain	D	P	D	M	0.648	34	0	2.81 × 10^−5^	0	Uncertain Significance	This study
c.1663C > T	15	p.P555S	Myosin head, motor domain	D	D	D	M	0.701	26.1	0	0	0	Uncertain Significance	This study
c.2648C > A	22	p.A883D	Myosin tail	D	D	D	M	0.692	28	0	0	0	Uncertain Significance	This study
c.3610G > A	28	p.E1204K	Myosin tail	T	B	D	L	0.678	24.7	0	2.29 × 10^−5^	0	Uncertain Significance	This study
c.4061T > C	31	p.L1354P	Myosin tail	D	D	D	M	0.813	28.4	0	0	0	Uncertain Significance	This study
c.5987_5993del		p.R1997Afs								0	0	0	Uncertain Significance	This study
c.[5991_5994del; 5996G > A]	43	p.[V1998Afs; R1999H]								0	0	0	Uncertain Significance	This study
				D	P	N	L	0.251	24.1	0	3.35 × 10^−5^	0	Uncertain Significance	This study
c.6003_6009del	43	p.F2002X								0	0	0	Uncertain Significance	This study

PP2, PolyPhen2; Mut Taster, Mutation Taster; Mut Assessor, Mutation Assessor; D, Deleterious (SIFT); B, Benign (SIFT); D, Probably Damaging (PP2); P, Possibly Damaging (PP2); A, Disease-causing automatic (Mut Taster); D, Disease-causing (Mutation Taster); N, Polymorphism (Mut Taster); M, Medium (Mut Assessor); L, Low (Mut Assessor).

**Table 3:** *MYH14* variants and clinical information in previous reports (all *MYH14* variants are indicated in NM_001145809.2).

Nucleotide Change	Amino Acid Change	Gender	Onset	Progression of HL	Age	Audiometric Configuration	PTA (Better-Hearing Ear)	Severity	Vestibular Symptoms	Intervention	Newborn Hearing Screening	Reference
c.20C > A	p.S7X	n/a	10 or 20	Yes	n/a	n/a	n/a	Severe to profound in 40 years	n/a	n/a	n/a	Donaudy et al., 2004 [3]
c.73C > T	p.Q25X	F	0 Y	Yes	7 M	n/a	n/a	n/a	n/a	n/a	n/a	Kim et al., 2017 [4]
		F	n/a	n/a	33 Y	Flat	82.5	Severe	n/a	n/a	n/a	
c.359C > T	p.S120L	n/a	n/a	n/a	28 Y	Flat	53.8	Moderate	n/a	n/a	n/a	Yang et al., 2005 [5]
		n/a	n/a	n/a	33 Y	Flat	65	Moderate	n/a	n/a	n/a	
		n/a	n/a	n/a	35 Y	Flat	76.3	Moderate	n/a	n/a	n/a	
		n/a	n/a	n/a	63 Y	Flat	72.5	Moderate	n/a	n/a	n/a	
c.505G > A	p.E169K	n/a	n/a	n/a	n/a	n/a	n/a	n/a	n/a	n/a	n/a	Sloan-Heggen et al., 2016 [8]
c.526G > A	p.A176T	n/a	n/a	n/a	n/a	n/a	n/a	n/a	n/a	n/a	n/a	Chen et al., 2016 [9]
c.541G > A	p.A181T	F	The first decade	n/a	n/a	U-sharped	73.75	Moderate	n/a	n/a	n/a	Qing et al., 2014 [10]
		M	The first decade	n/a	n/a	Flat	71.3	Moderate	n/a	n/a	n/a	
		F	The first decade	n/a	n/a	U-sharped	n/a	Severe	n/a	n/a	n/a	
c.572A > G	p.D191G	M	Congenital or prelingual	No?	5 Y	Flat	71.3	Moderate	n/a	n/a	n/a	Kim et al., 2017 [4]
c.823C > T	p.R275C	F	41 Y	Yes	44 Y	U-shaped	20	Mild	No	n/a	n/a	Iwasa et al., 2016 [6]
c.1049G > A	p.R350Q	n/a	n/a	n/a	n/a	n/a	n/a	n/a	n/a	n/a	n/a	Iwasa et al., 2016 [6]
c.1067C > T	p.T356M	n/a	n/a	n/a	n/a	n/a	n/a	n/a	n/a	n/a	n/a	Sommen et al., 2016 [11]
c.1150G > T	p.G384C	n/a	n/a	n/a	9 Y	n/a	n/a	Moderate	No	n/a	n/a	Donaudy., 2004 [3]
		n/a	n/a	n/a	n/a	n/a	n/a	n/a	n/a	n/a	n/a	Shearer et al.,2014 [12]
		n/a	n/a	n/a	n/a	n/a	n/a	n/a	n/a	n/a	n/a	Abouelhoda et al., 2016 [13]
c.1360G > A	p.A454T	n/a	n/a	n/a	n/a	n/a	n/a	n/a	n/a	n/a	n/a	Chen et al., 2016 [9]
c.1427G > A	p.R476H	n/a	n/a	n/a	n/a	n/a	n/a	n/a	n/a	n/a	n/a	Sloan-Heggen et al., 2016 [8]
c.1609G > A	p.D537N	F	n/a	Yes	8Y	Flat	40	Moderate	n/a	n/a	n/a	Kim et al., 2015 [14]
c.1625T > G	p.L542R	n/a	n/a	n/a	n/a	n/a	n/a	n/a	n/a	n/a	n/a	Sloan-Heggen et al., 2016 [8]
c.1919G > A	p.R640Q	n/a	n/a	n/a	n/a	n/a	n/a	n/a	n/a	n/a	n/a	Shearer et al., 2013 [7]
c.2089G > A	p.G697S	n/a	n/a	n/a	n/a	n/a	n/a	n/a	n/a	n/a	n/a	Iwasa et al., 2016 [6]
c.2203C> G	p.R735C	M	n/a	n/a	n/a	Flat	115	Severe	n/a	CI	n/a	Liu et al., 2019 [15]
		M	n/a	n/a	n/a	n/a	n/a	n/a	n/a	n/a	n/a	
c.2299C > A	p.R767S	n/a	n/a	Yes	n/a	n/a	n/a	Mild to moderate	No	n/a	n/a	Donaudy et al., 2004 [3]
c.2621T > C	p.L874P	n/a	n/a	n/a	n/a	n/a	n/a	n/a	n/a	n/a	n/a	Chen et al., 2016 [9]
c.2692A > C	p.K898Q	n/a	n/a	n/a	n/a	n/a	n/a	n/a	n/a	n/a	n/a	Miyagawa et al., 2013 [16]
c.2717C > T	p.T906M	F	The first decade	n/a	n/a	U-shaped	83	Severe	n/a	n/a	n/a	Qing et al., 2014 [10]
		F	The first decade	n/a	n/a	U-shaped	80	Severe	n/a	n/a	n/a	
c.2921G > A	p.R974H	n/a	n/a	n/a	n/a	n/a	n/a	n/a	n/a		n/a	Sloan-Heggen et al., 2016 [8]
c.2921G > T	p.R974L	M	n/a	n/a	52 Y	n/a	n/a	n/a	n/a	n/a	n/a	Choi et al., 2011 [17]
		M	n/a	n/a	48 Y	n/a	n/a	n/a	n/a	n/a	n/a	
		F	n/a	n/a	45 Y	n/a	n/a	n/a	n/a	n/a	n/a	
		F	n/a	n/a	41 Y	n/a	n/a	n/a	n/a	n/a	n/a	
		M	n/a	n/a	15 Y	n/a	n/a	n/a	n/a	n/a	n/a	
c.2921G > T	p.R974L	n/a	20 Y	Yes	n/a	n/a	n/a	n/a	n/a	n/a	n/a	Iyadurai et al., 2017 [20]
c.2921G > A	p.R974L	F	n/a	n/a	n/a	n/a	n/a	n/a	n/a	n/a	n/a	Almutawa et al., 2019 [21]
		F	n/a	n/a	58 Y	n/a	n/a	n/a	n/a	n/a	n/a	
		M	n/a	n/a	23 Y	n/a	n/a	n/a	n/a	n/a	n/a	
		F	n/a	n/a	24 Y	n/a	n/a	n/a	n/a	n/a	n/a	
c.3049C > T	p.L1017F	M	n/a	n/a	n/a	n/a	n/a	Mild to moderate	No	n/a	n/a	Donaudy et al., 2004 [3]
c.3877G > C	p.E1293Q	n/a	n/a	n/a	n/a	n/a	n/a	n/a	n/a	n/a	n/a	Sommen et al., 2016 [11]
c.4903G > A	p.E1635K	n/a	n/a	n/a	n/a	n/a	n/a	n/a	n/a	n/a	n/a	Miyagawa et al., 2013 [16]
c.5008C > T	p.R1670C	F	n/a	n/a	n/a	n/a	n/a	n/a	n/a	n/a	n/a	Vona et al., 2014 [19]
c.5020G > A	p.V1674M	n/a	n/a	n/a	n/a	n/a	n/a	n/a	n/a	n/a	n/a	Shearer et al., 2013 [7]
c.5176C > T	p.R1726W	n/a	n/a	n/a	n/a	n/a	n/a	n/a	n/a	n/a	n/a	Seco et al., 2017 [22]
c.5384G > A	p.R1795H	n/a	n/a	n/a	n/a	n/a	n/a	n/a	n/a	n/a	n/a	Moteki et al., 2017 [23]
c.5516C > A	p.A1839D	M	30 s	n/a	51 Y	Gently sloping	56.25	Moderate	No ^†^	n/a	n/a	Wang et al., 2020 [24]
		M	30 s	Yes	45 Y	Gently sloping	50	Moderate	n/a ^†^	n/a	n/a	
		M	10 s	Yes	29 Y	Flat	56.25	Moderate	No ^‡^	n/a	n/a	
c.5602G > A	p.A1868T	M	n/a	n/a	n/a	n/a	66.3	n/a	n/a	n/a	n/a	Kim et al., 2016 [18]

^†^ Low-amplitude in oVEMP; ^‡^ labyrinth reactivity lower in caloric test; n/a, not available; CI, Cochlear Implant.

## Data Availability

The sequencing data are available in the DDBJ databank of Japan (Accession number: JGAS000323).

## Supplementary Materials

The following are available online at https://www.mdpi.com/article/10.3390/genes12101623/s1, Table S1: The 68 deafness-causative genes analyzed in this study.
